# Plastidial wax ester biosynthesis as a tool to synthesize shorter and more saturated wax esters

**DOI:** 10.1186/s13068-021-02062-1

**Published:** 2021-12-15

**Authors:** Katharina Vollheyde, Ellen Hornung, Cornelia Herrfurth, Till Ischebeck, Ivo Feussner

**Affiliations:** 1 Department for Plant Biochemistry, Albrecht-von-Haller-Institute for Plant Sciences, University of Goettingen, Justus-von-Liebig-Weg 11, 37077 Goettingen, Germany; 2 Service Unit for Metabolomics and Lipidomics, Goettingen Center for Molecular Biosciences (GZMB), University of Goettingen, 37077 Goettingen, Germany; 3 Department for Plant Biochemistry, International Center for Advanced Studies of Energy Conversion (ICASEC) and Goettingen Center for Molecular Biosciences (GZMB), University of Goettingen, 37077 Goettingen, Germany

**Keywords:** *Arabidopsis thaliana*, Wax ester, Metabolic engineering, Wax synthase, Fatty acid reductase, *Marinobacter aquaeolei*

## Abstract

**Background:**

Wax esters (WE) are neutral lipids that consist of a fatty alcohol esterified to a fatty acid. WE are valuable feedstocks in industry for producing lubricants, coatings, and cosmetics. They can be produced chemically from fossil fuel or plant-derived triacylglycerol. As fossil fuel resources are finite, the synthesis of WE in transgenic plants may serve as an alternative source. As chain length and desaturation of the alcohol and acyl moieties determine the physicochemical properties of WE and their field of application, tightly controlled and tailor-made WE synthesis in plants would be a sustainable, beneficial, and valuable commodity. Here, we report the expression of ten combinations of WE producing transgenes in *Arabidopsis thaliana*. In order to study their suitability for WE production *in planta*, we analyzed WE amount and composition in the transgenic plants.

**Results:**

The transgenes consisted of different combinations of a *FATTY ACYL-COA/ACP REDUCTASE* (*FAR*) and two *WAX SYNTHASES/ACYL-COA:DIACYLGLYCEROL* O*-ACYLTRANSFERASES* (*WSD*), namely *WSD2* and *WSD5* from the bacterium *Marinobacter aquaeoleoi*. We generated constructs with and without plastidial transit peptides to access distinct alcohol and acyl substrate pools within *A. thaliana* cells. We observed WE formation with plastid and cytosol-localized FAR and WSD in seeds. A comparative WE analysis revealed the production of shorter and more saturated WE by plastid-localized WE biosynthesis compared to cytosolic WE synthesis.

**Conclusions:**

A shift of WE formation into seed plastids is a suitable approach for tailor-made WE production and can be used to synthesize WE that are mainly derived from mid- and long-chain saturated and monounsaturated substrates.

**Supplementary Information:**

The online version contains supplementary material available at 10.1186/s13068-021-02062-1.

## Background

Wax esters (WE) are in high demand for industrial applications. They are neutral lipids, and are composed of a fatty alcohol esterified to a fatty acid. The chain length and degree of unsaturation of incorporated alcohol and acyl moieties determine the physicochemical properties of WE [1]. Owing to their diverse physical and chemical properties, WE have a large range of industrial applications; they are used in inks, as coatings, for the production of candles, in cosmetics, or as lubricants, among other uses [2, 3]. In the past WE were primarily obtained from sperm whale, however they can now be synthesized chemically from fossil fuel, from plant-derived triacylglycerol (TAG) [4–6], or are extracted from seeds of *Simmondsia chinensis* [7, 8]. However, fossil fuel is a finite resource, and *S. chinensis* is challenging to cultivate [7, 8]. Therefore, WE production in transgenic plants has been discussed as a sustainable and inexpensive alternative solution [9–19].

Two key enzymes, fatty acyl-coenzyme A (CoA)/acyl carrier protein (ACP) reductases (FAR) and wax synthases (WS), are critical for the production of WE in plants. FAR synthesize fatty alcohols by the reduction of the carboxyl group of acyl-CoA/ACP, and WS catalyze the formation of WE from fatty alcohols and ACP- or CoA-activated fatty acids [20–23].

Up to now several combinations of FAR and WS enzymes have been expressed in *Arabidopsis thaliana*, *Camelina sativa*, *Crambe abyssinica*, *Brassica carinata*, *Lepidium campestre*, and *Nicotiana benthamiana* [9–19]. Studies have not only aimed for high WE amount, but also the synthesis of defined WE species that are desired for specific industrial applications. WE derived from monounsaturated long-chain substrates are particularly valuable in industry due to their excellent lubrication properties [9]. WE species synthesis in planta depends on the activities and substrate specificities of the expressed FAR and WS enzymes, and on the availability of acyl-CoA/ACP substrates. The combined expression of mouse FAR and mouse WS led to the formation of WE with mainly polyunsaturated 18 carbon acyl moieties in wild-type *A. thaliana* [9, 10]. Enzyme combinations of *Marinobacter aquaeolei* FAR (MaFAR) with jojoba WS (ScWS), *Acinetobacter baylyi* WSD1 (AbWSD1) or *M. aquaeolei* WSD5 (MaWSD5) produced WE with mainly monounsaturated 18 and 20 carbon acyl and alcohol moieties in wild-type *A. thaliana* [10, 13, 17]. Interestingly, expression of different enzyme combinations in the high oleic acid *A. thaliana* mutant *fad2 fae1* [24, 25] resulted in more than 60 mol% 18:1/18:1 (alcohol moiety/acyl moiety) WE [9, 10, 13]. The expression of condensing enzymes of the fatty acid elongation system in combination with WE producing enzymes led to the synthesis of longer WE in *B. carinata*, *C. sativa* and *L. campestre* [18, 19]. Shorter WE were generated in *C. sativa* upon co-expression of a 14:0 ACP thioesterase [12].

The above studies altered substrate availability through overexpression and knock out/down of fatty acid-synthesizing enzymes. These approaches may be disadvantageous in that they (i) interfere with the general fatty acid metabolism of the cell and (ii) additional genes have to be transformed into the WE producing plant. Interestingly, acyl-CoA/ACP biosynthesis per se gives rise to different acyl-CoA/ACP substrate pools within a plant cell [26, 27]. Plastids, the subcellular compartment of de novo fatty acid synthesis in plants, contain mainly 16:0 ACP, 18:0 ACP and 18:1 ACP. In contrast to that, cytosolic acyl molecules are bound to CoA and are more diverse in chain length and unsaturation as this pool is influenced by endoplasmic reticulum dependent acyl chain elongation and unsaturation processes. Taking advantage of the different substrate pools, we studied whether directing WE biosynthesis to plastids can be used as a tool to alter the formation of WE species. The work focused on the comparison of WE species synthesized by plastidial and cytosolic localized WS and FAR enzymes. Due to their lack of transmembrane domains affecting subcellular localization, we have chosen the bacterial *M. aquaeolei* MaFAR, MaWSD2, and MaWSD5 enzymes for this study. Combinations of the enzymes with and without plastidial transit peptides were generated and transformed into *A. thaliana*, and it was determined which WE species are synthesized in the different cellular compartments.

## Results

### Generation of transgenic *A. thaliana* plants expressing combinations of MaFAR, MaWSD2, and MaWSD5

To compare WE species synthesized from the plastidial and cytosolic fatty acid pools, we generated transgenic *A. thaliana* plants expressing different combinations of WE-forming enzymes with and without plastidial transit peptides. We chose to express FAR and WS enzymes from the bacterium *M. aquaeolei* (MaFAR [20, 22], MaWSD2 [17, 28, 29], MaWSD5 [17, 30]). Previous studies showed that MaWSD2 is a bifunctional WSD enzyme while MaWSD5 has only WS activity. Cytosolic expression of combinations of these enzymes resulted in WE production in planta [13, 17]. In addition, the lack of transmembrane domains in these bacterial enzymes avoids interference with the subcellular localization of the proteins.

**Fig. 1:**
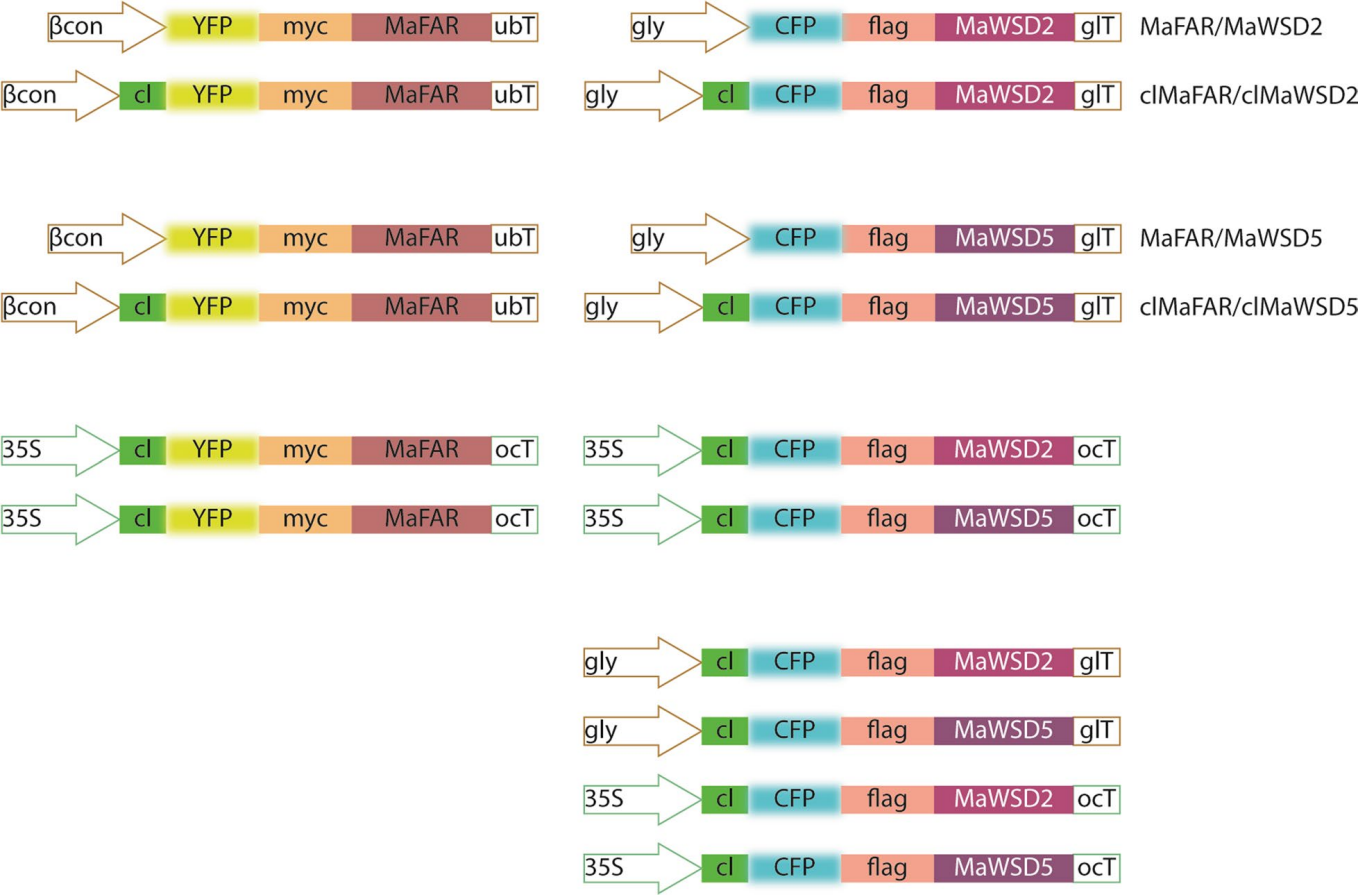
Constructs generated for *A. thaliana* transformation. The MaFAR/MaWSD5 construct was published recently [17]. βcon: β-conglycine promoter (*Glycine max*), seed specific; gly: glycinin promoter (*G. max*), seed specific; 35S: 35S promoter, ubiquitous promoter; ubT: ubiquitin 3 terminator; glT: glycinin terminator; ocT: octopine synthase terminator

To compare plastidial and cytosolic WE production we designed constructs following our recently published analysis of transgenic *A. thaliana* MaFAR/MaWSD5 plants [17] and generated nine additional constructs consisting of MaFAR combined with either MaWSD2 or MaWSD5 with and without plastidial transit peptides (Fig. 1; Table 1). All open reading frames were N-terminally fused with a fluorescence tag (yellow fluorescent protein (YFP) or cyan fluorescent protein (CFP)) and an epitope tag (myc or flag-tag) to facilitate localization studies and western blot analyses, respectively. Similar to the published and analyzed MaFAR/MaWSD5 (βcon::YFP-myc-MaFAR/gly::CFP-flag-MaWSD5) construct [17], a construct was created expressing YFP- and myc-tagged MaFAR under the control of the seed-specific β-conglycinin promoter and CFP- and flag-tagged MaWSD2 under the control of seed-specific glycinin promoter (βcon::YFP-myc-MaFAR/gly::CFP-flag-MaWSD2, from here on referred to as MaFAR/MaWSD2). In addition to these cytosolic enzyme combinations, another set of constructs was made by fusing the three enzymes with an N-terminal plastidial transit peptide [31] to re-direct WE biosynthesis from the cytosol to plastids, the location of de novo fatty acid biosynthesis (clMaFAR/clMaWSD2, clMaFAR/clMaWSD5). In order to confirm localization to plastids, two constructs were made aiming for a plastidial localization controlled by the 35S promoter (35S::clMaFAR/35S::clMaWSD2, 35S::clMaFAR/35S::clMaWSD5). The use of the 35S promoter facilitates ubiquitous gene expression and allows localization studies in green tissue in which plastids can be detected by chlorophyll autofluorescence. To analyze a possible contribution of plastidial fatty alcohols in WE production, a plastid-localized WSD alone was expressed by four more constructs (gly::clMaWSD2, gly::clMaWSD5, 35S::clMaWSD2, 35S::clMaWSD5).

**Table 1:** Overview of generated and analyzed transgenic *A. thaliana* plants

Construct expressed (short name) (βcon/gly: seed-specific promoters, 35S: 35S promoter)	Number of independent lines after herbicide treatment	Number of screened heterozygous lines by TLC (number of lines with increased WE amounts)	Plant lines used for further analysis
βcon::YFP-myc-MaFAR gly::CFP-flag-MaWSD2 (MaFAR/MaWSD2)	18	T2 seeds: 17 (9)	^‡§^lines 2, 6, 17
βcon::cl-YFP-myc-MaFAR gly::cl-CFP-flag-MaWSD2 (clMaFAR/clMaWSD2)	85	T2 seeds: 36 (22)	^‡§^lines 11, 28, 35
βcon::YFP-myc-MaFAR gly::CFP-flag-MaWSD5 (MaFAR/MaWSD5)	52	T2 seeds: 19 (16) [9 (6) lines screened for [17], 10 (10) additional lines screened for this publication]	^‡§^lines 11, 12, 17 (lines 2, 4, 5, 7, 10 [17])
βcon::cl-YFP-myc-MaFAR gly::cl-CFP-flag-MaWSD5 (clMaFAR/clMaWSD5)	43	T2 seeds: 18 (12)	^‡§^lines 4, 12, 18
35S::cl-YFP-myc-MaFAR 35S::cl-CFP-flag-MaWSD2 (35S::clMaFAR/35S::clMaWSD2)	72	T2 seeds: 22 (0) T1 leaves: 5 (0)	^†^line 21
35S::cl-YFP-myc-MaFAR 35S::cl-CFP-flag-MaWSD5 (35S::clMaFAR/35S::clMaWSD5)	56	T2 seeds: 23 (0) T1 leaves: 9 (0)	^†^lines 18, 19, 23
gly::cl-CFP-flag-MaWSD2	100	T2 seeds: 21 (0)	
gly::cl-CFP-flag-MaWSD5	95	T2 seeds: 21 (0)	
35S::cl-CFP-flag-MaWSD2	50	T2 seeds: 12 (0) T1 leaves: 5 (0)	
35S::cl-CFP-flag-MaWSD5	91	T2 seeds: 21 (0) T1 leaves: 38 (0)	

(^†^Confocal microscopy

^‡^Western blot

^§^Analysis of WE species and WE amounts by GC-FID and nanoESI-MS/MS)

Transformed *A. thaliana* Col-0 plants were first screened for independent transgenic T1 plants by herbicide treatment with glufosinate and 40 to 100 independent plants were obtained for each construct (Table 1), except for MaFAR/MaWSD2. Despite completing two transformations and screening a large number of seedlings, only 18 transgenic T1 plants were obtained for MaFAR/MaWSD2. For each construct, ca. 20 independent lines were screened for high WE content in T2 seeds by WE extraction and thin layer chromatography (TLC) [32]. For MaFAR/MaWSD5, ten new lines were screened in addition to the ones published by Vollheyde and colleagues [17]. In the case of MaFAR/MaWSD2, clMaFAR/clMaWSD2, MaFAR/MaWSD5 and clMaFAR/clMaWSD5 plants, 50–80% of the screened lines showed WE formation in T2 seeds. No WE formation was detected in T2 seeds of lines expressing plastid-localized enzymes under the control of the 35S promoter or expressing the plastid-localized MaWSD2 or MaWSD5 alone. As expression under the control of the 35S promoter should lead to ubiquitous expression of WE synthesizing enzymes, leaves of T1 plants expressing these constructs were screened for WE formation. However, no WE formation was detected by TLC analysis.

To examine whether plastidial localization of proteins was successful with the chloroplast transit peptide, 35S::clMaFAR/35S::clMaWSD2 and 35S::clMaFAR/35S::clMaWSD5 T2 seedlings were analyzed by confocal microscopy using the enzymes’ YFP and CFP tags (Fig. 1). Additional file 1 depicts CFP and YFP fluorescence overlay with chlorophyll autofluorescence, confirming plastidial localization of expressed clMaFAR and clMaWSD5. No CFP signal was obtained for 35S::clMFAR/35S::clMaWSD2.

As significant WE amounts were obtained in T2 seeds of plants expressing the four construct combinations MaFAR/MaWSD2, clMaFAR/clMaWSD2, MaFAR/MaWSD5 and clMaFAR/clMaWSD5, three independent lines per construct with high WE levels were chosen for further analyses of protein expression, WE total content, and WE composition (Table 1). For a comparison with results published for MaFAR/MaWSD5, three additional lines were analyzed in detail as the five already published ones were only analyzed by nanoelectrospray ionization tandem mass spectrometry (nanoESI-MS/MS) [17].

### MaFAR, MaWSD2 and MaWSD5 protein levels are different in seeds

**Fig. 2:**
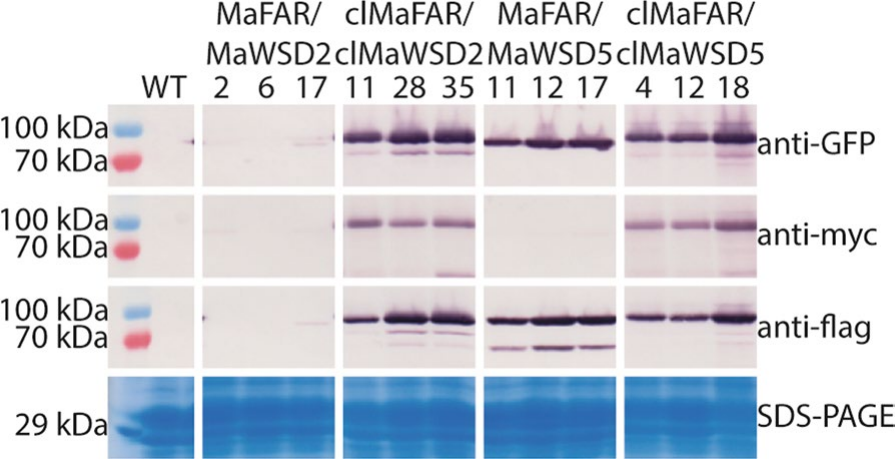
Western blot analysis of MaFAR/MaWSD2, clMaFAR/clMaWSD2, MaFAR/MaWSD5, and clMaFAR/clMaWSD5 seeds. Equal amounts of total T2 seed protein extracts were loaded on SDS gels for western blot analyses and SDS-PAGE. Protein detection was achieved by anti-GFP, anti-myc, and anti-flag IgG antibodies followed by the anti-Mouse IgG (whole molecule)-alkaline phosphatase. The SDS-PAGE gel, serving as loading control, was stained with coomassie. The experiment was performed once analyzing the three depicted independent plant lines per construct (MaFAR/MaWSD2: βcon::YFP-myc-MaFAR/gly::CFP-flag-MaWSD2, clMaFAR/clMaWSD2: βcon::cl-YFP-myc-MaFAR/gly::cl-CFP-flag-MaWSD2, MaFAR/MaWSD5: βcon::YFP-myc-MaFAR/gly::CFP-flag-MaWSD5, clMaFAR/clMaWSD5: βcon::cl-YFP-myc-MaFAR/gly::cl-CFP-flag-MaWSD5). For images of whole membranes and gel, see Additional file 2

Making use of their YFP-myc and CFP-flag tags, protein levels were investigated by western blot analysis in protein extracts of MaFAR/MaWSD2, clMaFAR/clMaWSD2, MaFAR/MaWSD5 and clMaFAR/clMaWSD5 dry T2 seeds. A detection of MaFAR was achieved via an anti-myc IgG antibody and a detection of MaWSD2 as well as MaWSD5 was done via an anti-flag IgG antibody. In addition, anti-green fluorescent protein (GFP) IgG antibody was used to monitor all three proteins via their YFP and CFP labels. MaFAR protein was detected in clMaFAR/clMaWSD2 and clMaFAR/clMaWSD5 constructs (Fig. 2, Additional file 2). Except for very weak signals in MaFAR/MaWSD2 lines 2 and 17, no signal corresponding to MaFAR protein was observed in MaFAR/MaWSD2 and MaFAR/MaWSD5 seeds. A signal corresponding to MaWSD5 protein was obtained in all MaFAR/MaWSD5 and clMaFAR/clMaWSD5 lines. In contrast to that, MaWSD2 protein was only detected in the clMaFAR/clMaWSD2 lines and as a weak signal in MaFAR/MaWSD2 line 17.

### Plastidial WE synthesis leads to a shift in WE length and desaturation degree

**Fig. 3:**
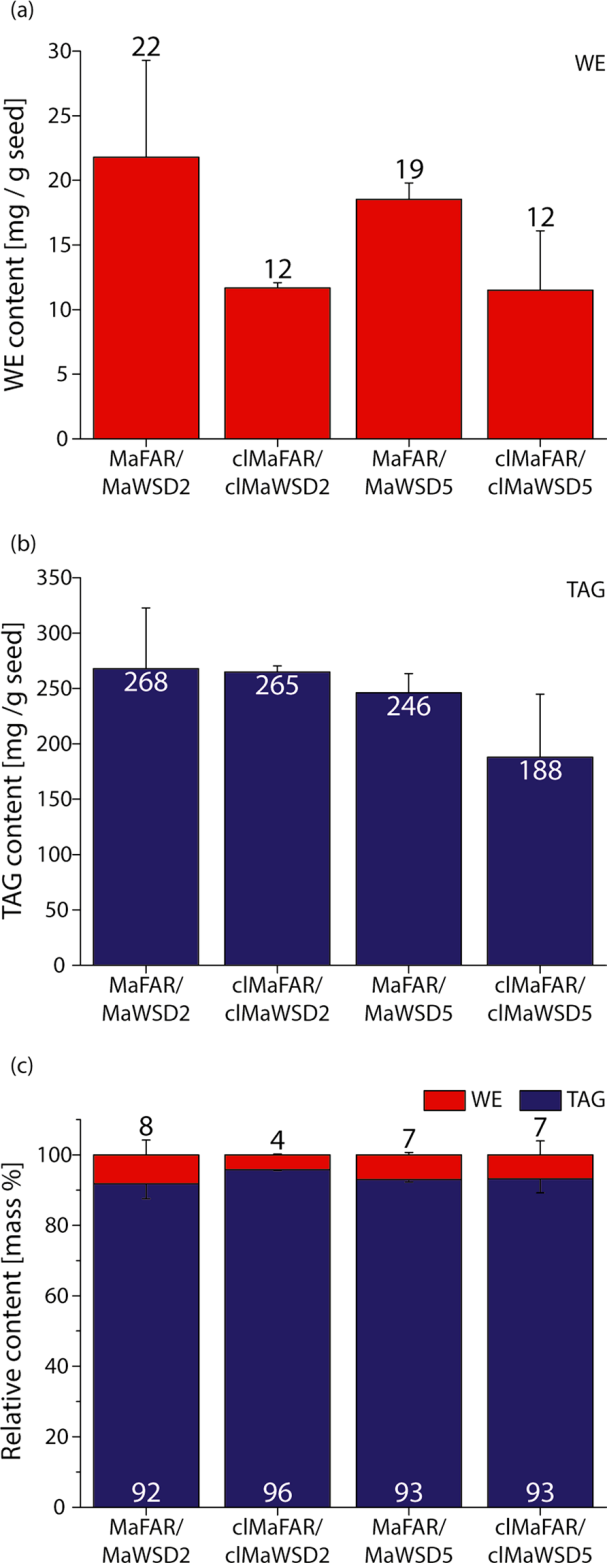
WE and TAG content of MaFAR/MaWSD2, clMaFAR/clMaWSD2, MaFAR/MaWSD5 and clMaFAR/clMaWSD5 seeds. Absolute WE (**a**) and TAG (**b**) amounts in mg/g seed were obtained by GC-FID analysis. Both values were used to calculate their relative content in mass% (**c**). Each bar represents the mean of three independent plant lines per construct determined in three extraction replicates (+ SD). Analysis of variance (ANOVA) revealed no significant differences in absolute and relative WE and TAG contents between the constructs. For the data from each plant line, see Additional file 3. The raw data are provided in Additional file 4. Analyzed wild-type *A. thaliana* seeds contained 323 mg TAG/g seed (mean of three extraction replicates, SD: 17 mg TAG/g seed)

In order to determine WE amount and species generated by the four constructs (MaFAR/MaWSD2, clMaFAR/clMaWSD2, MaFAR/MaWSD5 and clMaFAR/clMaWSD5), T2 seeds were analyzed by gas chromatography coupled to flame ionization detection (GC-FID) and nanoESI-MS/MS. GC-FID analysis revealed WE contents between 12 and 22 mg/g seed and TAG contents between 188 and 268 mg/g seed (Fig. 3, Additional files 3 and 4). Although differences in WE levels were not significant between the different constructs, seeds expressing clMaFAR/clMaWSD2 contained on average ~ 50% less WE than MaFAR/MaWSD2 seeds. In clMaFAR/clMaWSD5 seeds, the averaged WE content was ~ 60% of the WE amount of MaFAR/MaWSD5 seeds (Fig. 3a). Similar to WE content, no significant difference in TAG content was observed in seeds between the constructs (Fig. 3b). However, MaFAR/MaWSD5 TAG content was slightly reduced compared to seeds expressing MaWSD2 and in clMaFAR/clMaWSD5 seeds, the TAG content was reduced even more. Figure 3c shows that MaFAR/MaWSD2 seeds contained on average 8% WE, as a fraction of the total storage lipids, which was even higher in an individual line (Additional file 3c). In clMaFAR/clMaWSD2 lines, the percentage of WE was 4%. Due to accompanied changes in total TAG amount, WE content in MaFAR/MaWSD5 and clMaFAR/clMaWSD5 seeds accounted for 7%.

**Fig. 4:**
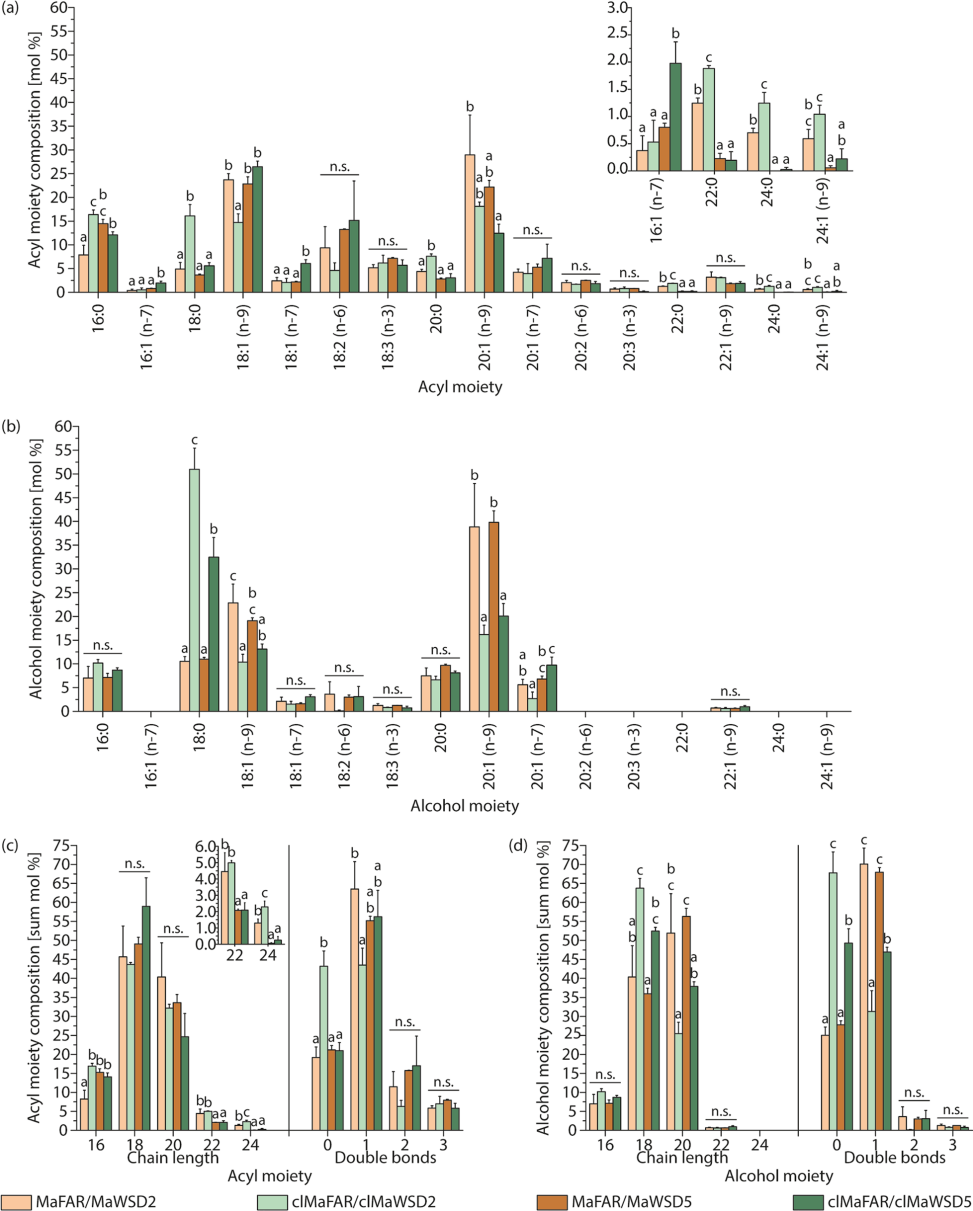
Acyl and alcohol moiety profiles of seed WE from MaFAR/MaWSD2, clMaFAR/clMaWSD2, MaFAR/MaWSD5 and clMaFAR/clMaWSD5. Acyl (**a**) and alcohol (**b**) moiety profiles were obtained by GC-FID analysis. Relative abundances of WE moieties are displayed in mol%. Combined relative abundances of acyl (**c**) and alcohol (**d**) moieties with similar chain length or desaturation degree were attained by summing up relative abundances of respective moieties. Each bar represents the mean of three independent plant lines per construct determined in three extraction replicates (+ SD). For better visibility minor fatty acids showing significant changes are shown as insets with a different scale. ANOVA analysis followed by post hoc Tukey test was performed separately for each acyl and alcohol moiety as well as each chain length and double bond number (n.s.: not significant). For p-values of the ANOVA analysis, see Additional file 6. For the data from each plant line, see Additional file 5. The raw data are provided in Additional file 4

In order to analyze whether plastidial localization of WE-producing enzymes leads to changes in generated WE species, their fatty acid and fatty alcohol profiles were further investigated by GC-FID (Fig. 4, Additional files 4, 5 and 6). The acyl moiety profile shows only small differences between MaFAR/MaWSD2 and MaFAR/MaWSD5. Both enzyme combinations led to WE consisting mostly of 20:1 (n-9), 18:1 (n-9), 18:2 (n-6) and 16:0 fatty acids (Fig. 4a). For MaFAR/MaWSD5 a significantly greater content of 16:0 was detected.

Comparing MaFAR/MaWSD2 and clMaFAR/clMaWSD2, plastidial WE biosynthesis led to a significantly reduced incorporation of 18:1 (n-9) accompanied with a greater content of 18:0 and 16:0 acyl moieties (Fig. 4a). Although not significant, a trend towards reduced amounts of WE with 20:1 (n-9) and 18:2 (n-6) acyl moieties was observed as well. Comparing MaFAR/MaWSD5 and clMaFAR/clMaWSD5, reduced 20:1 (n-9) and 16:0 content was observed with the plastidial constructs, even though these differences were not significant. This was accompanied by an increase in 18:0 and 18:1 (n-9), as well as a significantly greater 18:1 (n-7) acyl moiety content.

The overall chain length and desaturation of acyl moieties reflects the above-mentioned trends. For both plastidial constructs a decrease in 20 carbon acyl moieties compared to the corresponding non-plastidial constructs was detected, although this decrease was not significant. Whereas the decrease in 20 carbon chain length species resulted mainly in a significant increase in 16 carbon acyl moieties for clMaFAR/clMaWSD2, acyl moieties with 18 carbons chain length increased in clMaFAR/clMaWSD5 seed WE (Fig. 4c). The number of double bonds present in acyl moieties did not differ between MaFAR/MaWSD5 and clMaFAR/clMaWSD5. In contrast to that, a clear and significant trend towards the incorporation of saturated acyl moieties was observed for clMaFAR/clMaWSD2 compared to the corresponding non-plastidial construct. While monounsaturated acyl species were favored by the non-plastidial construct, saturated and monounsaturated acyl moieties were equally distributed in clMaFAR/clMaWSD2.

Figure 4b shows the alcohol moiety profiles of extracted WE. No differences were observed between MaFAR/MaWSD2 and MaFAR/MaWSD5. In both enzyme combinations 20:1 (n-9) and 18:1 (n-9) were the preferred alcohol species incorporated into WE. Comparing the alcohol profiles of plastidial and corresponding non-plastidial constructs, a clear and significant decrease in 20:1 (n-9) alcohol species to almost half of the content was observed, as well as a decrease in 18:1 (n-9). This was accompanied with a large and significant increase in 18:0 alcohol moiety as well as a slight, although not significant, increase in 16:0 in both plastidial constructs. Interestingly, in clMaFAR/clMaWSD2 the 18:0 alcohol moiety content was significantly more than in clMaFAR/clMaWSD5.

Figure 4d shows the summed up overall chain length and desaturation degree preference for alcohol moieties of WE in the analyzed lines. Whereas alcohol moieties with 20 carbons were preferred over 18 carbons in non-plastidial constructs, the incorporation of fatty alcohols with 18 carbons was preferred with plastidial constructs. A slight, although not significant, increase in 16 carbon alcohol species was observed in the same combinations as well in comparison to the corresponding non-plastidial constructs. A large shift occurred in the number of double bonds. MaFAR/MaWSD2 and MaFAR/MaWSD5 preferred monounsaturated alcohol moieties with ~ 70 mol%. In both plastidial enzyme combinations, the number of double bonds decreased significantly in alcohol moieties. In clMaFAR/clMaWSD5 saturated and monounsaturated species accounted to equal amounts of ~ 50 mol%. In clMaFAR/clMaWSD2, the content of saturated and monounsaturated alcohol moieties even inverted compared to MaFAR/MaWSD2 accounting for ~ 70 mol% saturated moieties in the plastidial construct.

**Fig. 5:**
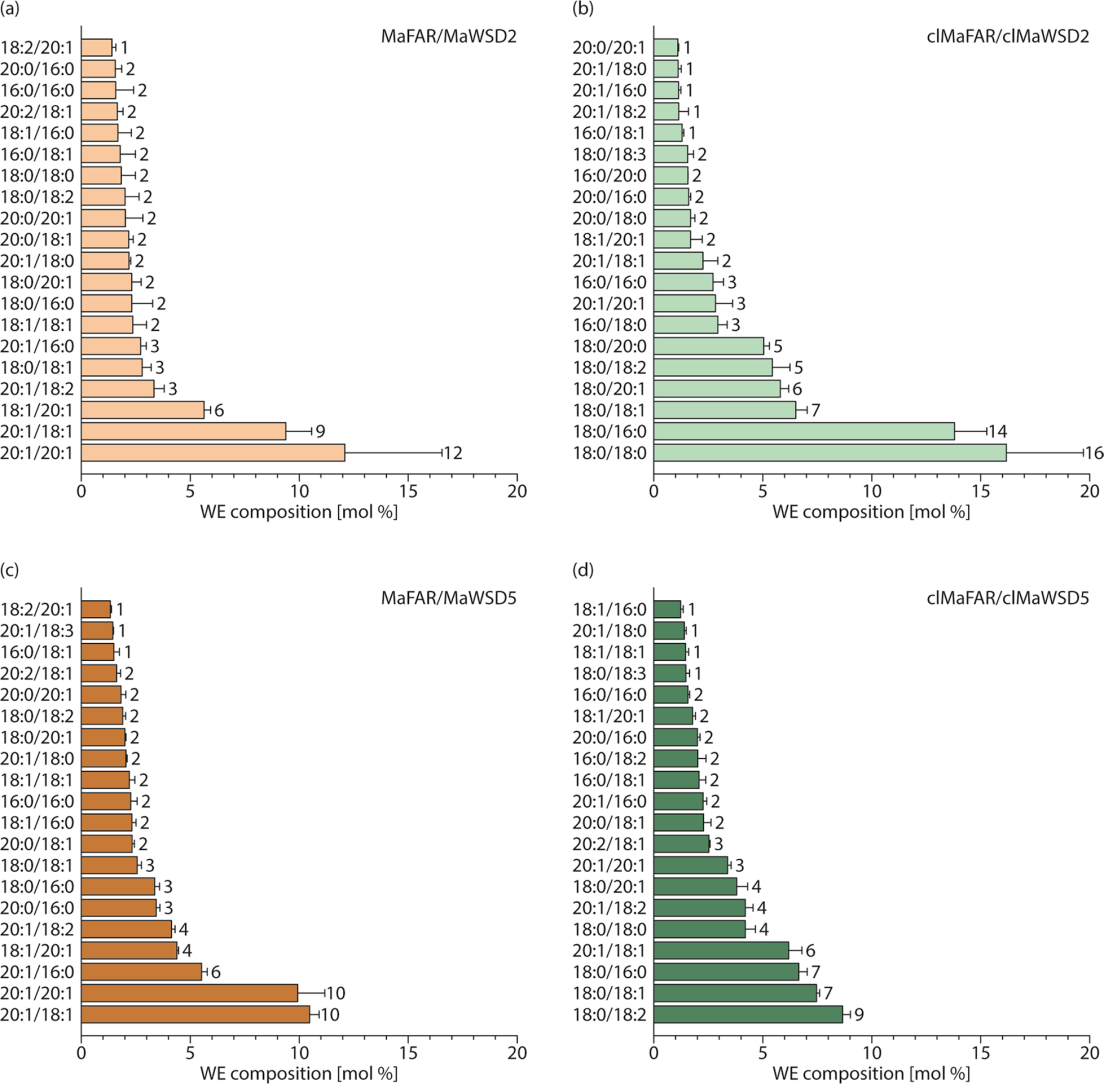
Analysis of seed WE species in MaFAR/MaWSD2, clMaFAR/clMaWSD2, MaFAR/MaWSD5 and clMaFAR/clMaWSD5. Molecular WE species were analyzed by nanoESI-MS/MS in MaFAR/MaWSD2 (**a**), clMaFAR/clMaWSD2 (**b**), MaFAR/MaWSD5 (**c**) and clMaFAR/clMaWSD5 (**d**) T2 seeds. Displayed are relative abundances of the top 20 WE species (alcohol moiety/acid moiety) of each construct in mol%. Each bar represents the mean of three independent plant lines per construct determined in three measuring replicates (+ SD). The raw data are provided in Additional file 7

Acyl and alcohol moiety profiles obtained by GC-FID analysis provide an overview of the composition of acyl and alcohol species of WE. However, information about individual, entire WE species cannot be obtained by this analysis. Therefore, WE of three independent plant lines per construct were analyzed by nanoESI-MS/MS (Fig. 5, Additional file 7). Figure 5 shows the 20 most abundant WE species synthesized by the four analyzed enzyme combinations. As already observed in the GC-FID profiles, seeds expressing either MaFAR/MaWSD2 or MaFAR/MaWSD5 have similar WE composition. In both constructs, 20:1/18:1 and 20:1/20:1 were the two most abundant WE species, which accounted for ~ 20 mol%. In clMaFAR/clMaWSD2 seeds, however, more than 50 mol% of all WE species contained 18:0 alcohol moieties, and were components of the six most abundant WE species. 18:0/18:0 and 18:0/16:0 were the two main WE species in clMaFAR/clMaWSD2 seeds, accounting for 30 mol%. Similar to but not as consistent as in clMaFAR/clMaWSD2 seeds, 18:0 alcohol moieties were preferred by clMaFAR/clMaWSD5 accounting for ~ 30 mol%. 18:1 and 18:2 acyl moieties formed the two most abundant WE species in clMaFAR/clMaWSD5.

## Discussion

Previous studies suggest that the acyl-CoA pool is the primary determinant of the composition of WE in seeds. Therefore, we tested how shifting WE synthesis from the cytosol to the plastid affects the WE profile. We expressed combinations of *M. aquaeolei* FAR and WSD enzymes in *A. thaliana* with and without plastidial transit peptides and analyzed which WE species are generated by the enzyme combinations in different cellular compartments. The bacterial enzymes were chosen as they lack transmembrane domains that might interfere with subcellular protein localization, and because they were previously shown to be able to synthesize WE in *A. thaliana* [6, 19].

We detected WE in plants expressing MaFAR and MaWSD combinations in seeds with both cytosolic and plastid-localized enzymes. Combined GC-FID and nanoESI-MS/MS analyses revealed the expected shift towards shorter and more saturated WE species synthesized by enzymes localized in plastids (Figs. 4 and 5). Seeds of transgenic MaFAR/MaWSD2 and MaFAR/MaWSD5 contained WE with equal amounts of 20:1 (n-9) and 18:1 (n-9) acyl moieties accounting for 20–25 mol% each, and to a lesser extent with 18:2 (n-6) and 16:0 acyl moieties, accounting for 10–15 mol% each (Fig. 4). As a fatty alcohol moiety, 20:1 (n-9) was favored by both enzyme combinations followed by 18:1 (n-9) (Fig. 4). In contrast to this, seeds expressing the plastidial constructs contained WE with predominantly 18:0 alcohol moieties accounting for 30–50 mol% (Fig. 4) and the clMaFAR/clMaWSD2 seeds accumulated WE with a higher content of 16:0 and 18:0 acyl moieties compared to MaFAR/MaWSD2 seeds (Fig. 4). These results show that a direction of WE biosynthesis to seed plastids can be used as a tool to alter substrate availability for tailor-made WE production. This approach can be used as an alternative to overexpression or downregulation/knocking out of fatty acid modifying enzymes, which was done in the past to alter substrate availability [9, 10, 12, 13, 18, 19]. An additional benefit of this approach was that there was no significant reduction in WE amount when WE biosynthesis was redirected from the cytosol to seed plastids (Fig. 3). The total WE amounts we obtained (12 to 22 mg/g seed (Fig. 3)) are comparable with WE contents obtained previously upon expression of bacterial FAR and WS enzymes in *A. thaliana*. 4—17 mg/g seed were obtained with constructs expressing MaFAR with different bacterial WSD enzymes [13] and 4.7—19.8 mg/g seed for MaFAR/MaWSD5 lines [17].

Although not significant, a slight trend towards lower WE amount upon plastidial WE biosynthesis compared to cytosolic WE formation was observed (Fig. 3). This might be caused by insufficient WE storage capacity in plastids. Co-expression with plastidial FAR/WSD of structural proteins that coat the WE-storing lipid droplets, or plastoglobuli in the plastids, may increase the yield of WE. Another reason for lower WE amounts of seeds expressing plastidial constructs might be a lack of acyl-ACP availability for WE biosynthesis due to efficient fatty acid usage by competing pathways such as export or synthesis of other lipids. Aslan and colleagues [14] reported that an increase in fatty acid biosynthesis does not lead to an increase in plastidial WE amount. Co-expression of different plastidial FAR/WS constructs in *N. benthamiana* together with the *A. thaliana* transcription factor AtWRI1, described to induce de novo fatty acid synthesis in plastids [33, 34], increased WE formation only for one of the tested enzyme combinations [14]. In order to increase acyl-ACP availability for plastidial WE biosynthesis, the downregulation of competing pathways might be necessary, but has to be done carefully; plastid-synthesized fatty acids are building blocks for the whole lipid complement of a plant cell. Hence, a total block of competing acyl-ACP metabolism cannot be achieved. A third explanation for lower WE content in the plastid-targeted lines might be counter-selection for high expression of the plastidial constructs during as suggested by another study. The authors of this study observed markedly lower WE amounts in stable transformed *N. benthamiana* plants compared to transient transformed ones when expressing a fusion construct consisting of a transit peptide, MaFAR, and *Marinobacter hydrocarbonoclasticus* WS2 under the control of the 35S promoter [14, 15]. They observed that surviving plants showed stunted growth and chlorotic leaves and stems and assumed a counter-selection for high construct expression during the regeneration process.

When expressing plastid-localized enzymes under the control of the 35S promoter instead of a seed-specific promoter, we did not observe detectable WE formation in leaves and seeds. This observation may be explained by a counter-selection for high plastidial WE content as well. Plastidial WE amounts might be more harmful in certain developmental stages than in others. They might be tolerated during later stages of seed development, but detrimental during early seedling development. It would be interesting to determine seedling lethality of plants expressing plastid-localized WE biosynthesis enzymes under the control of the 35S promoter compared to the same proteins under the control of seed-specific promoters. However, as screening for transgenic plants was performed in this study through herbicide resistance, a detailed seedling lethality rate was difficult to determine and not analyzed here.

The two most abundant acyl and alcohol moieties in WE from the transgenic MaFAR/MaWSD2 and MaFAR/MaWSD5 seeds analyzed here are 20:1 (n-9) and 18:1 (n-9) moieties (Fig. 4). This preference was also observed in MaFAR/MaWSD5 plants analyzed previously [17]. In contrast to that, Yu D, Hornung E, Iven T and Feussner I [13] published acyl and alcohol profiles of seed WE produced by a combination of MaFAR and MaWSD2 with the two most abundant acyl moieties being 18:0 and 18:1 and the two most abundant alcohol moieties being 18:1 and 18:2. It has to be noted that in the publication by Vollheyde and colleagues [17] the same MaFAR/MaWSD5 constructs were expressed as here, whereas Yu and colleagues [13] expressed MaFAR and MaWSD2 without additional YFP-myc/CPF-flag-tags and under the control of the seed-specific napin promoter instead of β-conglycinin and glycinin promoters. The preference for shorter substrates by the MaFAR/MaWSD2 combination described by Yu and colleagues [13] compared to the MaFAR/MaWSD2 combination published here may be explained by the different promotors that were chosen to regulate expression of the enzymes. The time of promoter activity during seed development might be different for the napin promoter compared to the β-conglycinin and the glycinin promoters. Baud and colleagues [35] reported that the fatty acid profile changes during the development of *A. thaliana* seeds. While until torpedo stage around 50% of all seed fatty acids are 16:0 and 18:0 fatty acids, the content of both fatty acids decreases and the amount of 18:3 and 20:1 fatty acids increases during the course of seed development. This results in different acyl substrate pools available for WE biosynthesis over time during seed development. Another explanation for differences in WE acyl and alcohol moiety profiles of MaFAR/MaWSD2 constructs between this study and the work of Yu and colleagues [13] might be the presence of N-terminal YFP-myc and CFP-flag fusions in the constructs analyzed here, which could influence the substrate specificities of the proteins. In addition to that, differences in expression levels might be a third explanation, which were not tested by Yu and colleagues [13].

## Conclusions

A demand for sustainable tailor-made WE production is increasing as fossil fuel resources are finite. Here, we report the analysis of transgenic *A. thaliana* plants expressing ten different enzyme combinations of the bacterial MaFAR, MaWSD2 and MaWSD5. Detailed lipid analysis revealed that redirection of WE formation to plastids in seeds is possible. The availability of acyl-ACP with altered chain length and desaturation degree compared to acyl-CoA present in the cytosolic substrate pool resulted in the production of shorter and more saturated WE in plastids compared to the cytosol. Consequently, the study presented here shows that redirection of WE biosynthesis to seed plastids is a powerful tool to alter substrate availability for tailor-made WE production in plants.

## Methods

### Generation of transgenic *A. thaliana* plants

Transgenic *A. thaliana* plants were generated according to Vollheyde and colleagues [17]. Using Gateway technology (Thermo Fisher Scientific) binary transformation vectors were generated for simultaneous transformation of two enzymes as described previously [9].

Using fusion polymerase chain reaction, several constructs were generated from sequence combinations of *Escherichia coli* codon optimized MaFAR (Accession Number: WP_011785687.1), MaWSD2 (Accession Number: ABM20141.1), MaWSD5 (Accession Number: ABM20482.1), YFP, CFP, myc-tag, flag-tag, and a plastidial localization sequence corresponding to an 80 amino acid signal peptide [31]. As fusion polymerase chain reaction was not successful for generating constructs containing MaWSD2, these constructs were made by classical cloning via an *Apa*I restriction site on the 5´ end of the MaWSD2 sequence. Generated constructs were cloned into desired pENTRY vectors (pENTRYA carrying a 35S promoter, pENTRYB carrying a β-conglycinin promoter, pENTRYC carrying a glycinin promotor, pENTRYD carrying a 35S promotor) via *Sal*I/*Bam*HI restriction sites or *Xho*I/*Bgl*II restriction sites for MaWSD5 containing constructs. In total, nine pENTRY vectors were produced. Using Gateway technology (Thermo Fisher Scientific) ten binary vectors were generated from combinations of the destination vector (pCAMBIA33) together with either a combination of pENTRYB, pENTRYC and an empty pENTRYA vector or with a combination of pENTRYA (empty one in case only a WSD will be expressed) and pENTRYD vector. Primer sequences can be found in Additional file 8.

### Screening of transgenic *A. thaliana* plants

Screening of transgenic *A. thaliana* plants by analysis of seed WE was performed as described previously [17].

For the screening of transgenic *A. thaliana* plants by leaf WE, three leaves were harvested per plant, pooled and lyophilized. For rapid screening, WE extraction was performed in 2 mL microtubes. 500 µL methanol was added to lyophilized leaf material and the samples were shaken for 20 min at 4 °C. Afterwards, 1 mL hexane was added to each sample and samples were shaken for 15 min at 4 °C. After centrifugation (5 min 11,360 g), the upper hexane phase was transferred to a 1.5-mL microtube. Subsequent to hexane evaporation in a Savant SPD131DDA SpeedVac Concentrator (Thermo Scientific) with a Savant RVT5105 Refrigerated Vapor Trap (Thermo Fisher Scientific), extracted lipids were dissolved in 50 µL chloroform and spotted on a TLC silica plate (TLC Silica gel 60, 20 × 20 cm, Merck Millipore). The TLC plate was developed with hexane/diethyl ether/acetic acid (90:10:1, *v/v/v*) as running solvent, which yielded best results in separating WE and carotenoids. Bands of neutral lipids were visualized by dipping the plate into a CuSO_4_ solution (10% (*w/v*) CuSO_4_, 6.8% (*v/v*) phosphoric acid) and subsequent heating of the plate to 190 °C.

### Analysis of WE and TAG by GC-FID

Lipid extraction, sample preparation and GC-FID analysis of WE and TAG was performed as described previously [10].

### Analysis of WE species by nanoESI-MS/MS

WE analysis was performed by nanoESI-MS/MS with a 6500 QTRAP® tandem mass spectrometer (AB Sciex) as previously described [32].

### Western blot

Proteins were extracted from frozen and homogenized seeds. For 4 mg seed material, 100 µL freshly prepared extraction buffer (4% (*w/v*) SDS, 2% (*v/v*) β-mercaptoethanol, 2 mM phenylmethane sulfonyl fluoride, 0.1 M Tris pH 8.5) was added. Samples were immediately, vigorously vortexed for at least 2 min. Afterwards, the samples were incubated at 80 °C for 3 min and centrifuged (10 min, 20,810 g, room temperature). The supernatant was transferred to a new tube and was mixed with 4 × Läemmli buffer. For SDS-PAGE and western blot analysis, 10 µL of with 4 × Läemmli buffer diluted protein extract was loaded on an SDS gel. For western blot analysis, proteins were detected using an anti-GFP antibody (diluted 1:5,000, BioLegend), monoclonal anti-c-MYC antibody (1:5000, Sigma) and monoclonal anti-FLAG M2 antibody (1:5,000, Merck) followed by the anti-Mouse IgG (whole molecule)-alkaline phosphatase (diluted 1:30,000, Merck). The SDS gel serving as loading control was stained with coomassie.

### Microscopy

Seedlings were grown on ½ MS agar plates containing 1% (*w/v*) sucrose for 3 days under long day condition (16 h light, 8 h darkness, 22 °C) subsequent to 2–3 days of stratification.

Images were recorded using a Zeiss LSM 780 confocal microscope (Carl Zeiss Inc., Jena, Germany). eCFP was excited at 458 nm and detected at a wavelength of 462–520 nm imaged using a T80/R20 beam splitter, or at 463–510 nm using a MBS 458 beam splitter; eYFP was excited at 514 nm and detected at a wavelength of 523–622 nm imaged using a T80/R20 beam splitter, or at 515–551 nm using a MBS 458/514 beam splitter; chlorophyll was excited at 633 nm and detected at a wavelength of 647–722 nm imaged using a T80/R20 beam splitter, or at 647–721 nm using a MBS 488/561/633 beam splitter. Images of 35S::clMaFAR/35S::clMaWSD5 lines 18, 19 and 23 (upper image) were recorded with the settings described first. Images of 35S::clMaFAR/35S::clMaWSD2 line 21 and 35S::clMaFAR/35S::clMaWSD5 line 23 (lower image) were recorded using the settings described second. Pictures were processed with Image J 1.50i [36].

### Statistical analysis

Statistical analysis of WE content and profile was performed using ANOVA analysis and post hoc Tukey test. The analysis of the profiles was performed separately for each acyl and alcohol moiety as well as each chain length and double bond number. The p-values of the ANOVA analysis are depicted in Additional file 6. The post hoc Tukey test was conducted using the glht-function of the R multcomp library.

## Supplementary Information

**Additional file 1**: Localization studies of WE-producing enzymes in seedlings using confocal microscopy. Pictures were taken from transgenic 35S::clMaFAR/35S::clMaWSD2 (35S::cl-YFP-myc-MaFAR/35S::cl-CFP-flag-MaWSD2) and 35S::clMaFAR/35S::clMaWSD5 (35S::cl-YFP-myc-MaFAR/35S::cl-CFP-flag-MaWSD5) T2 seedlings. Pictures were processed with Image J 1.50i [36]. The scale bar represents 6 µm.

**Additional file 2**: Western blot analysis of MaFAR/MaWSD2, clMaFAR/clMaWSD2, MaFAR/MaWSD5, and clMaFAR/clMaWSD5 seeds. Equal amounts of total T2 seed protein extracts were loaded on SDS gels for western blot analyses and SDS-PAGE. Protein detection was achieved with anti-GFP, anti-myc and anti-flag IgG antibodies followed by the anti-Mouse IgG (whole molecule)—Alkaline Phosphatase. The SDS-PAGE gel, serving as loading control, was stained with coomassie. The experiment was performed once analyzing the three depicted independent plant lines per construct (MaFAR/MaWSD2: βcon::YFP-myc-MaFAR/gly::CFP-flag-MaWSD2, clMaFAR/clMaWSD2: βcon::cl-YFP-myc-MaFAR/gly::cl-CFP-flag-MaWSD2, MaFAR/MaWSD5: βcon::YFP-myc-MaFAR/gly::CFP-flag-MaWSD5, clMaFAR/clMaWSD5: βcon::cl-YFP-myc-MaFAR/gly::cl-CFP-flag-MaWSD5).

**Additional file 3**: WE and TAG content of MaFAR/MaWSD2, clMaFAR/clMaWSD2, MaFAR/MaWSD5, and clMaFAR/clMaWSD5 seeds. Absolute WE (a) and TAG (b) amounts in mg/g seed were obtained by GC-FID analysis. Both values were used to calculate their relative content in mass% (c). Each bar represents the mean of three extraction replicates (+SD).

**Additional file 4**: Raw data GC-FID analysis. The document contains raw data connected to Fig. 3, Fig. 4, Additional file 3, and Additional file 5.

**Additional file 5**: Acyl and alcohol moiety profiles of seed WE from MaFAR/MaWSD2, clMaFAR/clMaWSD2, MaFAR/MaWSD5 and clMaFAR/clMaWSD5. Acyl (a) and alcohol (b) moiety profiles were obtained by GC-FID analysis. Displayed are relative abundances of WE moieties in mol% of three independent plant lines per construct. Each bar represents the mean of three extraction replicates (+SD).

**Additional file 6**: p-Values of the ANOVA analysis shown in Fig. 4.

**Additional file 7**: Raw data nanoESI-MS/MS analysis. The document contains data connected to Fig. 5.

**Additional file 8**: Primer sequences.

## Data Availability

All data generated or analyzed during this study are included in this published article and its supplementary information files. IF is responsible for distribution of materials presented in this article.

## Abbreviations

ACP: Acyl carrier protein
CFP: Cyan fluorescent protein, CoA: coenzyme A
FAR: Fatty acyl-CoA/ACP reductase
GC-FID: Gas chromatography coupled to flame ionization detection
GFP: Green fluorescent protein
nanoESI-MS/MS: Nanoelectrospray ionization tandem mass spectrometry
TAG: Triacylglycerol
TLC: Thin layer chromatography, WE: wax ester
WS: Wax synthase
WSD: Wax synthases/acyl-CoA:diacylglycerol *O*-acyltransferases
YFP: Yellow fluorescent protein
