# Supplementary figures and images for: Plastidial wax ester biosynthesis as a tool to synthesize shorter and more saturated wax esters
Source: Biotechnol Biofuels. 2021 Dec 15;14:238. doi: 10.1186/s13068-021-02062-1 (PMC8675476; doi:10.1186/s13068-021-02062-1)

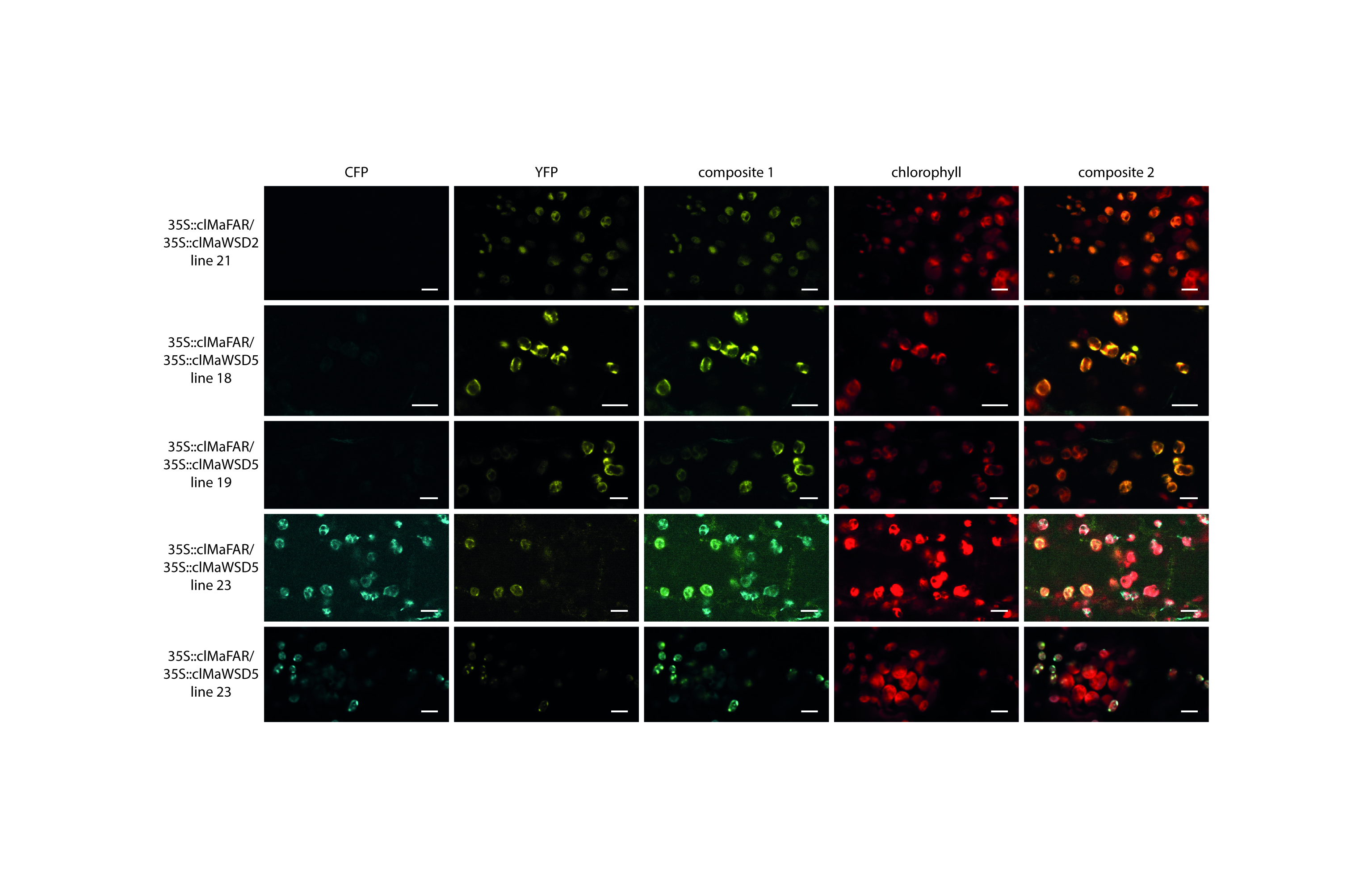

Supplement: Supplementary file 1 — Additional file 1: Localization studies of WE-producing enzymes in seedlings using confocal microscopy. Pictures were taken from transgenic 35S::clMaFAR/35S::clMaWSD2 (35S::cl-YFP-myc-MaFAR/35S::cl-CFP-flag-MaWSD2) and 35S::clMaFAR/35S::clMaWSD5 (35S::cl-YFP-myc-MaFAR/35S::cl-CFP-flag-MaWSD5) T2 seedlings. Pictures were processed with Image J 1.50i [36]. The scale bar represents 6 µm. [file 13068_2021_2062_MOESM1_ESM.tif]

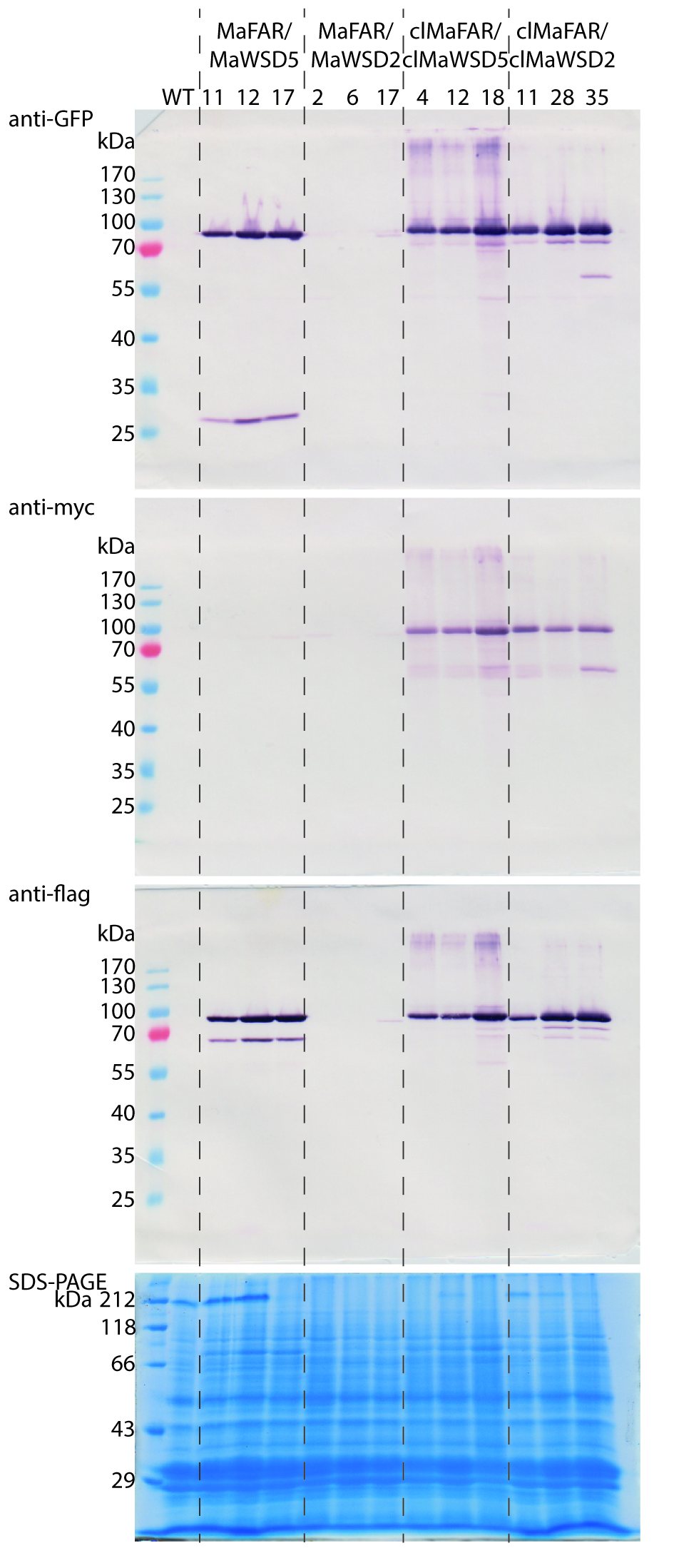

Supplement: Supplementary file 2 — Additional file 2: Western blot analysis of MaFAR/MaWSD2, clMaFAR/clMaWSD2, MaFAR/MaWSD5, and clMaFAR/clMaWSD5 seeds. Equal amounts of total T2 seed protein extracts were loaded on SDS gels for western blot analyses and SDS-PAGE. Protein detection was achieved with anti-GFP, anti-myc and anti-flag IgG antibodies followed by the anti-Mouse IgG (whole molecule)—Alkaline Phosphatase. The SDS-PAGE gel, serving as loading control, was stained with coomassie. The experiment was performed once analyzing the three depicted independent plant lines per construct (MaFAR/MaWSD2: βcon::YFP-myc-MaFAR/gly::CFP-flag-MaWSD2, clMaFAR/clMaWSD2: βcon::cl-YFP-myc-MaFAR/gly::cl-CFP-flag-MaWSD2, MaFAR/MaWSD5: βcon::YFP-myc-MaFAR/gly::CFP-flag-MaWSD5, clMaFAR/clMaWSD5: βcon::cl-YFP-myc-MaFAR/gly::cl-CFP-flag-MaWSD5). [file 13068_2021_2062_MOESM2_ESM.tif]

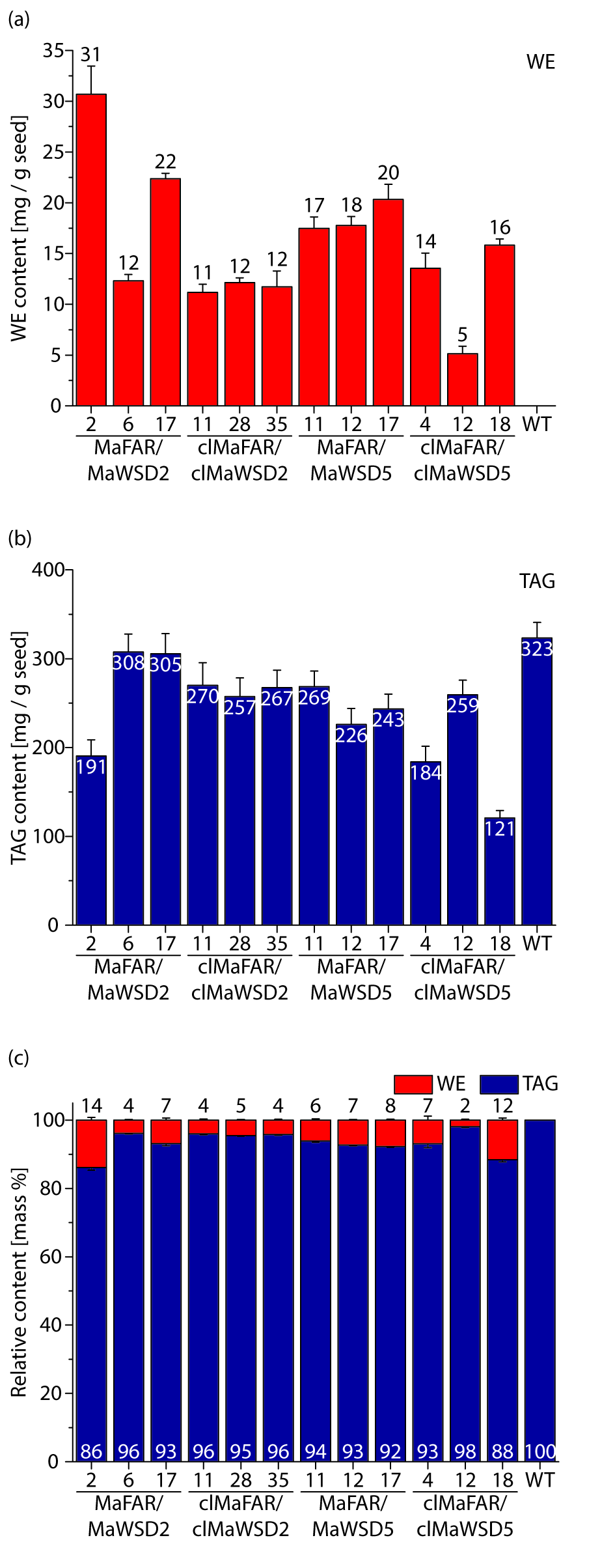

Supplement: Supplementary file 3 — Additional file 3: WE and TAG content of MaFAR/MaWSD2, clMaFAR/clMaWSD2, MaFAR/MaWSD5, and clMaFAR/clMaWSD5 seeds. Absolute WE (a) and TAG (b) amounts in mg/g seed were obtained by GC-FID analysis. Both values were used to calculate their relative content in mass% (c). Each bar represents the mean of three extraction replicates (+SD). [file 13068_2021_2062_MOESM3_ESM.tif]

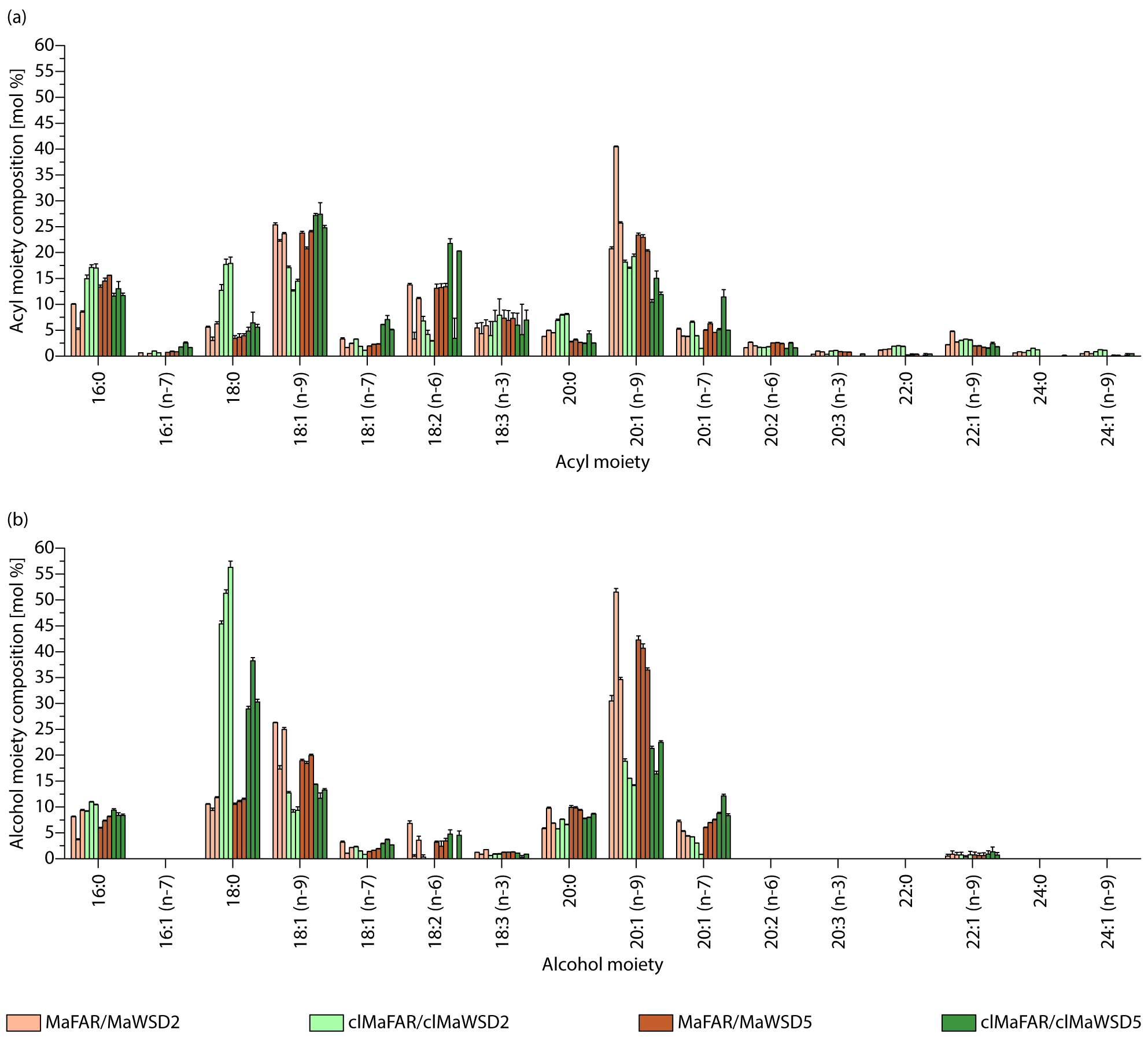

Supplement: Supplementary file 5 — Additional file 5: Acyl and alcohol moiety profiles of seed WE from MaFAR/MaWSD2, clMaFAR/clMaWSD2, MaFAR/MaWSD5 and clMaFAR/clMaWSD5. Acyl (a) and alcohol (b) moiety profiles were obtained by GC-FID analysis. Displayed are relative abundances of WE moieties in mol% of three independent plant lines per construct. Each bar represents the mean of three extraction replicates (+SD). [file 13068_2021_2062_MOESM5_ESM.tif]
