# Supplementary material for: Plastidial wax ester biosynthesis as a tool to synthesize shorter and more saturated wax esters
Source: Biotechnol Biofuels. 2021 Dec 15;14:238. doi: 10.1186/s13068-021-02062-1 (PMC8675476; doi:10.1186/s13068-021-02062-1)
Supplement: Supplementary file 6 — Additional file 6: p-Values of the ANOVA analysis shown in Fig. 4. [file 13068_2021_2062_MOESM6_ESM.docx]

**Additional file 6** ANOVA analysis p-values. *** p<0.001; ** p<0.01; * p<0.05.

| **Figure** | **Sample name** | **ANOVA p-value** |
| --- | --- | --- |
| **Acyl moiety**  **profile**  **(Fig. 4a)** | 16:0 | ******* |
|  | 16:1 (n-7) | ****** |
|  | 18:0 | ******* |
|  | 18:1 (n-9) | ******* |
|  | 18:1 (n-7) | ****** |
|  | 18:2 (n-6) | **not significant** |
|  | 18:3 (n-3) | **not significant** |
|  | 20:0 | ******* |
|  | 20:1 (n-9) | ***** |
|  | 20:1 (n-7) | **not significant** |
|  | 20:2 (n-6) | **not significant** |
|  | 20:3 (n-3) | **not significant** |
|  | 22:0 | ******* |
|  | 22:1 (n-9) | **not significant** |
|  | 24:0 | ******* |
|  | 24:1 (n-9) | ******* |
| **Alcohol moiety**  **profile**  **(Fig. 4b)** | 16:0 | **not significant** |
|  | 18:0 | ******* |
|  | 18:1 (n-9) | ****** |
|  | 18:1 (n-7) | **not significant** |
|  | 18:2 (n-6) | **not significant** |
|  | 18:3 (n-3) | **not significant** |
|  | 20:0 | **not significant** |
|  | 20:1 (n-9) | ****** |
|  | 20:1 (n-7) | ****** |
|  | 22:1 (n-9) | **not significant** |
| **Acyl moiety**  **chain length, double bonds**  **(Fig. 4c)** | C16 | ****** |
|  | C18 | **not significant** |
|  | C20 | **not significant** |
|  | C22 | ****** |
|  | C24 | ******* |
|  | DB0 | ******* |
|  | DB1 | ***** |
|  | DB2 | **not significant** |
|  | DB3 | **not significant** |
| **Alcohol moiety**  **chain length, double bonds**  **(Fig. 4d)** | C16 | **not significant** |
|  | C18 | ******* |
|  | C20 | ****** |
|  | C22 | **not significant** |
|  | DB0 | ******* |
|  | DB1 | ******* |
|  | DB2 | **not significant** |
|  | DB3 | **not significant** |
