# Supplementary material for: Plastidial wax ester biosynthesis as a tool to synthesize shorter and more saturated wax esters
Source: Biotechnol Biofuels. 2021 Dec 15;14:238. doi: 10.1186/s13068-021-02062-1 (PMC8675476; doi:10.1186/s13068-021-02062-1)
Supplement: Supplementary file 8 — Additional file 8: Primer sequences. [file 13068_2021_2062_MOESM8_ESM.docx]

**Additional file 8** Primer sequences.

| Primer Name | Primer Sequence (5`-> 3`) |
| --- | --- |
| cl-for-*Sal*I | ACT**GTCGAC**ATGGCTTCCTCTATGCTCTCTTC |
| YFP/CFP-for-*Sal*I | ACT**GTCGAC**ATGGTGAGCAAGGGCGAGGAG |
| MaFAR-rev-*Bam*HI | GC**GGATCC**TCATGCCGCTTTTTTACG |
| MaWSD2-rev-*Bam*HI | GC**GGATCC**TTACTTGCGGGTTCGGGCGCGC |
| MaWSD2-for-*Apa*I | AGC**GGGCCC**ATGAAACGTCTCGGAACCCTGG |
| Flag-rev-*Apa*I | AGT**GGGCCC**CTTATCGTCGTCATCCTTGTAATCC |
| cl-for-*Xho*I | ACT**CTCGAG**ATGGCTTCCTCTATGCTCTCTTC |
| CFP-for-*Xho*I | ACT**CTCGAG**ATGGTGAGCAAGGGCGAGGAG |
| MaWSD5-rev-*Bgl*II | GC**AGATCT**TCAGTCCAGCTGATCCAGTTCCGC |
